# Editorial: Natural Products-Based Drugs: Potential Therapeutics Against Alzheimer’s Disease and Other Neurological Disorders

**DOI:** 10.3389/fphar.2019.01417

**Published:** 2019-11-26

**Authors:** Muhammad Ayaz, Farhat Ullah, Abdul Sadiq, Myeong Ok Kim, Tahir Ali

**Affiliations:** ^1^Department of Pharmacy, University of Malakand, Chakdara, Pakistan; ^2^Division of Applied Life Science (BK 21), College of Natural Science, Gyeongsang National University, Jinju, South Korea; ^3^Department of Comparative Biology and Experimental Medicine, University of Calgary, Alberta, Canada

**Keywords:** Alzheimer’s disease, natural products, cognition, phytochemicals, β-amyloid

Alzheimer’s disease (AD) and dementia are disorders of the aging population and becoming major health care burden worldwide due to unavailability of complete therapy. AD is the most frequent cause of dementia among 60% to 80% patients and has effected 45 million people globally which is estimated to triple by 2050 (Alzheimer’s, 2015). AD is a progressive, neurodegenerative disorder, characterized by behavioral turbulence, cognitive dysfunctions, imperfection in routine life activities, thus putting a huge socioeconomic burden on the health care system (Ahmad et al., 2015; Ali et al., 2017; Ayaz et al., 2017b). Among the pathophysiological hallmarks of the disease are the deficiency of vital neurotransmitter acetylcholine (ACh), deposition of amyloid plaques (Aβ), highly phosphorylated tau proteins, and imbalance in gluatamatergic system (Ayaz et al., 2017a; Khalil et al., 2018; Ovais et al., 2018a). Only five drugs are clinically approved for use, among which tacrine, galantamine, donepezil, and rivastigmine are cholinesterase inhibitors whereas the fifth one memantine is glutamatergic system modulator (Ayaz et al., 2015; Kamal et al., 2015). These drugs have limited efficacy and are associated with side effects like tacrine is hepatotoxic (Watkins et al., 1994). Currently, results from clinical trials performed in mild to moderate AD dementia have directed researchers to find more effective yet safe alternatives from natural sources (Yiannopoulou and Papageorgiou, 2013; Cummings et al., 2014; Ovais et al., 2018b).

The plant kingdom consists of a huge number of species with tremendous diversity of bioactive metabolites with different chemical scaffold (Ramawat et al., 2009; Ahmad et al., 2016; Mir et al., 2019). According to reports, only 6% and 15% of medicinal plants have been systematically investigated for pharmacological and phytochemical potentials respectively (Choudhary, 2001). Since, natural products are synthesized by living organisms, they have naturally optimized properties for various biological functions including binding to specific bimolecules or target proteins. Comparison of the structural features of natural and compounds synthetic revealed that the major difference between the two sources originates from starting points which makes synthesis more easy. For instance, separation of chiral compounds is a big challenge, so usually molecules with less number of chiral centers is synthesized and favored (Jan et al., 2019; Hussain et al., 2019). Besides the less number of chiral centers, synthetic molecules have low molecular weight, high chain lengths, less number of Lipinski type H-bond receptors and donors, less oxygen, and more halogen, nitrogen and sulfer. Moreover, synthetic compounds have a high number of freely rotatable bonds, low number of rings, complex ring systems, high octanol-H_2_O_2_ partition coefficients (cLogP), and high degree of saturation. Consequently, due to less number of chiral centers and the abovementioned properties make the synthetic compounds less specific for biological targets. In contrast, natural compounds own selective biological properties due to affinity for specific proteins, have superior chemical diversity and biosynthetic complexity and more beneficial ADME/T properties (JI and ZHANG, 2008; Atanasov et al., 2015). Of particular interest regarding drug discovery is the use of ethnomedicinally important plants which are already proven to be safe and effective in human populations. This classical knowledge-based approach includes observation, description, and experimental investigations on bioactive metabolites. This approach is more effective, for instance, in the evaluation of 122 plant-derived compounds approved for clinical use as drugs, 80% originated from ethnomedicinal use of the same in local population for the same disease.

Considering the “One-compound multiple-targets paradigm” for the development of more effective anti-AD drugs, natural compounds have got special interest. Despite partial success of the synthetic agents as potential multifunctional anti-AD drugs, the pharmacokinetics and safety issues are their major limiting factors (Fink et al., 1996). In contrast, natural compounds originated from medicinal plants or dietary sources have proven efficacy on multiple targets with broad safety profiles. For instance, curcumin has been reported to ameliorate cognitive dysfunction symptoms *via* modulation of inflammatory pathways in central nervous system, decline in free radicals load, chelate metals ions, inhibit Aβ aggregation, and thus is a potential multi-potent anti-AD candidate (Frautschy et al., 2001; Lim et al., 2001; Baum and Ng, 2004; Ono et al., 2004; Yang et al., 2005). Other flavonoids, including catechins, gossypetin, and myricetin, are also potential pleiotropic anti-AD agents, as they restrain Aβ aggregation, inhibit vital enzymes, and scavenge free radicals (Rice-Evans et al., 1996; Ayaz et al., 2019). Structure-activity relationship (SAR) studies on the flavonoids suggested that catechol moiety is a vital pharmacophore responsible for the anti-oxidant, anti-amyloid potentials of these compounds (Lashuel et al., 2002; Zhang, 2005). These findings suggest the development of catechol-based, multi-potent anti-AD drugs.

Ethnopharmacology, a source of knowledge-driven drug discovery is playing a significant role in drug discovery from plants, animals, and fungi based on local or traditional knowledge of its pharmacological or toxicological properties in local population (Cordell and Colvard, 2005; Heinrich et al., 2009; Heinrich, 2010). Currently, about 119 drugs approved for clinical use are derived from medicinal plants. Among these, 74% were discovered from chemical identification of the constituents responsible for medicinal use by humans. These 119 drugs derived from plants are commercially produced from <90 species of plants. As there are more than 25000 species on the globe, their systematic analysis can lead to the development of more useful drugs against various diseases (Farnsworth, 1990). For the development of pharmaceuticals ranging from digitalis to vincristine, ethnopharmacological approach of drug discovery is proven extremely successful. The advent of high throughput, mechanisms-based bioassays coupled with plants candidates derived from painstaking ethnopharmacological research has lead to the discovery of novel pharmaceuticals almost in all major groups of drugs. The most important step in the drug discovery from natural sources is the selection of most suitable starting materials based on ethnobotanical, ethnomedicinal, and folkloric uses. Ethnopharmacological knowledge aid in drug discovery by providing three basic levels of information: 1. “As general indicator of non-specific bioactivity suitable for a panel of broad screens” 2. “As an indicator of specific bioactivity suitable for particular high-resolution bioassays” 3. “As an indicator of pharmacological activity for which mechanism-based bioassays have yet to be developed” (Cox, 1994).

Galanthamine, a cholinesterase inhibitor is widely distributed alkaloid in several species of Amaryllidaceae family. The discovery and development of this modern drug for the management of AD is based on ethnopharmacological knowledge of its use in Europe (Heinrich and Teoh, 2004). The alkaloid was initially isolated from snowdrop (*Galanthus* spp., particulary *Galanthus woronowii* Losinsk.), and now obtained from several members of the same family including snowflake (*Leucojum* spp., particularly *Leucojum aestivum* L.), daffodil (*Narcissus* spp.) as well as from synthetic sources. The historical development of galanthamine till its approval for clinical use is comprised of several phases (Heinrich, 2010). According to some unconfirmed reports by a Bulgarian pharmacologist, people were applying common snowdrop on their foreheads to relieve nerve pain (Mashkovsky and Kruglikova-Lvova, 1951). Russian pharmacologists reported that in the early 1950s local villagers living at the foot of Ural mountains used wild Caucasian snowdrop for the treatment of disease in children they considered to be poliomyelitis (Shellard, 2000). In 1951, the first ever anti-cholinergic study on galanthamine was reported by Mashkovsky and Kruglikova-Lvova using rat smooth muscles (Heinrich, 2010). In 1952, Proskurnina and Yakovleva published the chemical structure of galanthamine isolated from *G. woronowii* (Paskov, 1986). In 1955, Mashkovsky published yet another cholinesterase inhibitory study on galanthamine but the source of galanthamine used was not reported. In 1956, Bulgarian pharmacologist D. Paskov reported the discovery of galanthamine from European daffodil and snowdrop, *Galanthus nivalis*. In 1960, an in-vivo cholinesterase inhibitory study was reported on galanthamine and in 1980s researchers working on AD started investigations of its therapeutic effects in detail. In 1990s, ganthamine was developed for clinical use and Sanochemia Pharmazeutika obtained the patency rights of galanthamine in 1996. In 2000, galanthamine was licensed for treatment of AD in UK, Iceland, Ireland, and Sweden. By now, it is used globally for the symptomatic relief of the AD. Unfortunately, galanthamine has limited efficacy and only delay the onset of severe symptoms but offer no complete eradication of the disease (Heinrich, 2010).

Physostigmine also known as eserine is another alkaloid isolated from the calaabar bean *Physostigma venenosum* Balf. in 1864 (Mach et al., 2004). Physostigmine was used as anti-glaucoma drug for the first time in 1877 (Howes and Perry, 2011). And importantly, it was the first discovered AChE inhibitor which provided a foundation for the discovery and use in clinical conditions in 1980s. Owing to the presence of carbamate moiety it is a useful cholinesterase inhibitor and is used in glaucoma, AD, myasthenia gravis, and atropine-induced coma (Stilson et al., 2001). Despite of efficacy as AChE inhibitor, physostigmine has serious limitations including short half-life (30 min), narrow therapeutic index, gastrointestinal side effects it is not in clinical practice for the management of neurological disorders (Giacobini et al., 1987). However, the chemical structure of physostigmine provided a template for the development of more useful AChE inhibitors including rivastigmine (Orhan and Senol, 2013). Rivastigmine was licensed for clinical use in UK as a remedy in symptomatic relief of mild to moderate AD. Thus, these plant-derived alkaloids and AChE inhibitors are useful agents for the development drugs for the management neurological disorders (Griffith, 2008).

This special topic was a platform for relevant experts in the field of ethnopharmacology and neuropharmacology to share cutting edge research and emerging literature-based reviews related to AD and other neurological disorders. The main objective of this research topic was to consider research and reviews related to the potential development of new drugs from natural sources against AD. A sufficient number of submissions focused on prevention to therapy of AD and other neurological disorders were considered. Gaiardo et al. reported the expression and possible role of dorsal hippocampus proteins in the memory enhancing properties of the standardized *Ginkgo biloba* extract in animal models. Authors used proteomic analysis to study the effect of *G. biloba* therapy on dorsal hippocampus proteins expression pattern which regulate CREB activity and synaptic plasticity implicated in long-term memory formation. *G. biloba* therapy at various doses was found to aid in retention of original memory, effect proteins involved in remodeling of cytoskeleton, size, shape, and stability of dendritic spines and formation of myelin sheath. Thus, *G. biloba* therapy modulates long-term memory *via* differential proteins expression which might act as important target in cognitive dysfunction disorders. *G. biloba* leaves extracts from different sources were also reported to rescued animals’ brain against Aβ_42_-induced neurotoxicity and electrophysiological alterations (Bader et al.). In a literature review, Javed et al. reported the inhibitory effects of phytochemicals on a pre-synaptic regulatory protein “α-Synuclein.” Literature clearly links the aggregation, oligomerization, and fibrillation of α-Synuclein with Parkinson’s disease, and inhibition of these processes is among the key strategies to counteract the disease. Plant extracts and isolated compounds were found to inhibit α-Synuclein fibril formation or aggregation and might be effective remedies against Parkinsonism related synucleinopathies .

Owing to the significance of cholinesterase inhibitors therapy in AD, dos Santos et al. summarized the potential role of plant based cholinesterase inhibitors as lead anti-AD agents. Diverse group of extracts and phytochemicals including polyphenolics, alkaloids terpenes, and coumarins from 54 plant species and 29 families were evaluated. Alkaloids were found to be the most promising cholinesterase inhibitors, which required further studies including SAR analysis.

Several authors employed scopolamine-induced AD model to check the neuroprotective effects of plants extracts and isolated compounds. Embelin an active constituent of *Embelia ribes* fruit and previously known cholinesterase inhibitor was tested by Bhuvanendran et al. for its anti-amnesic and nootropic effects in rat model at 0.3 to 1.2 mg/kg doses for 17 days. Cognitive defects were induced by 1 mg/kg of scopolamine for 9 days and the effects of embelin on cognition was assessed *via* elevated plus maze, novel object recognition paradigm. Moreover, gene expression for BDNF, CREB_1_, and mRNS levels of antioxidant enzymes (CAT, SOD_1_) were checked in hippocampus tissues of the animals. Sub-chronic treatment with embelin significantly improved recognition index and memory retention in behavioral models and increased inflection ratio in nootropic assay. Further, embelin increased the expression of BDNF, CREB_1_, CAT, SOD_1_ genes, and inhibited neurochemical and histological changes in scopolamine induced AD model. Using the same model, Zhou et al. studied the protective effect of seed extract from *Moringa oleifera*. Cognitive impairment was induced by 4 mg/kg i/p injection of scopolamine for six days in mice. Pretreatment with oral 250 to 500 mg/kg of *M. oleifera* significantly ameliorated scopolamine mediated cognitive dysfunction and improved cholinergic system reactivity and neurogenesis. Further, *M. oleifera* revived the proteins expressions for CREB, ERK_1/2_, Akt suppressed by scopolamine therapy, suggesting its beneficial effects are mediated *via* improvement of cholinergic neurotransmission and activation of vital signaling pathways. In another study by Mushtaq et al. the methanolic extract of *Lavandula stoechas* L considerably improved cognitive performance of rodents using elevated plus maze, light and dark, and hole board models. *L. stoechas* therapy improved the activity of antioxidant enzymes including CAT, SOD, GSH in the brain and reduced MDA, AChE activity in the brain tissues.

Quercetin a widely distributed natural flavonoid was evaluated by Khan et al. for its protective effect against lipopolysaccharide (LPS) induced neuroinflammatory and neuro-protective potentials. Quercetin therapy at 30 mg/kg for 2 weeks considerably reduced activated gliosis, markers of inflammation, and neuroinflammatory process in cortex and hippocampus of mice brain. Further, it prevented mitochondrial apoptosis and neurodegeneration *via* regulation of Bax/Bcl2, declining cytochrome-c activation, caspase-3 activity, and breakdown of PARP-1 in cortex and hippocampus. Quercetin therapy significantly improved cognitive performance and upturned LPS-induced neuronal loss in animal brain.

In a systematic review Ma et al. considered the neurocognitive potentials of traditionally important plant *Rhodiola rosea* L. Review included 36 studies and concluded that *R. rosea* improve cognitive performance in animals models *via* regulation of cholinergic neurotransmission, improving coronary blood flow, decline in neuro-inflammation, apoptosis, and free radicals load. Zhang et al. evaluated Da-Bu-Yin-Wan a Chinese herbal medicine for its ameliorative effects on DJ-1 protein-associated mitochondrial dysfunctions and Akt signaling in rat adrenal pheochromocytoma cells (PC-12). The PC-12 cells were transfected with plasmid pcDNA3-Flag-DJ-1 and were subsequently exposed to 1-methyl-4-phenyl pyridinium (Parkinsonism-related mitochondrial toxin) in the presence and absence of test sample. In Da-Bu-Yin-Wan-treated groups, the mitochondrial toxin-induced toxicity was significantly reduced, and DJ-1 expression was increased. Further, Akt phosphorylation was increased by DJ-1 expression. Thus, Da-Bu-Yin-Wan improved the ameliorative effects of DJ-1 on mitochondrial dysfunction *via* increasing Akt phosphorylation. In another study, gintonin, a ginseng-derived lysophosphatidic receptor ligand was reported for its neuroprotective effects in 1-methyl-4-phenyl-1,2,3,6-tetrahydropyridine induced neurotoxicity model of Parkinson disease. Pre-treatment of animals with 100 mg/kg of gintonin considerably reduced motor dysfunctions, reduced loss of tyrosine hydroxylase-positive neurons, and inhibited activation of microglia and expression of inflammatory mediators following MPTP injection. Gintonin therapy also blocked MAPKs, NF-kB pathways, and activated Nrf2, LPARs pathways post MPTP injection (Choi et al., 2018).

Taraxasterol isolated from *Taraxacum officinale* was reported by Liu et al. for its anti-neuroinflammatory potentials in LPS-stimulated BV2 micoglial cells. Taraxasterol considerably reduced LPS-mediated TNF-α, IL-1β generation, and activation of NF-kB. It has dislocated lipids rafts formation and prevented TLR4 translocation into lipids rafts. Moreover, taraxasterol has activated LXRα-ABCA1 signaling pathway which cause induction of cholesterol efflux from cells, concluding that it inhibits LPS-mediated neuroinflammatory process in microglia cells *via* activation of LXRα-ABCA1 signaling pathway. The role of phytochemicals as anti-neuroinflammatory agents in AD were summarized by Shal et al. in a comprehensive review. They concluded that plant derived secondary metabolites including flavonoids, phenolic derivatives, saponins, glycosides, alkaloids, and terpenoids mediate their neuroprotective effects *via* reduction of excessive microglial activation, expression of cytokines, NF-kβ, and ROS burden. Ullah and Khan evaluated the published literature on silymarin isolated from *Silybum marianum* in the context of its anti-Parkinson’s potentials. Silymarin was concluded to mediate its anti-Parkinson therapeutic effects *via* decline in oxidative stress, inflammatory cytokines, and alteration of cellular apoptosis, estrogen receptor machinery.

Yet in another research study, fatty acids rich extract from *Clerodendrum volubile* was reported to restrain cell migration, decline oxidative stress, and regulates cell cycle progression in glioblastoma multiforme (U87MG) cells (Erukainure et al.). Owing to the role molecular simulation studies in drug discovery, Rasool et al. performed molecular docking studies on albiziasaponin-A, orientin, salvadorin against AChE, COX2, and MMP8 proteins. All compounds exhibited strong interactions with the target proteins with lowest binding energies comparable to already approved drugs. In-vivo studies suggested that these compounds considerably declined oxidative stress and inflammatory markers in serum of Sprague Dawley rate model of AD (Rasool et al., 2018). In an anti-depressant and anxiolytic study, two compounds isolated from ethyl acetate fraction of *Quercus incana* showed beneficial anxiolytic effects using Elevated Plus Maze and Light and Dark paradigms. In the presence of flumazenil (selective benzodiazepine receptor antagonist), the anxiolytic activity of the test compounds were reduced, suggesting that benzodiazepine binding site of GABA-A receptors might be involved in this activity. Further, both compounds exhibited significant anti-depressant potentials in force swimming and tail suspension tests (Sarwar et al.).

In conclusion, medicinal plants are a major source of diverse bioactive constituents. Ethnopharmacology-directed studies will not only provide scientific base for the effective dose, potential toxicological effects to local community but can lead to the development of more effective multi-target drugs for the prevention and treatment of various diseases including neurological disorders.

## Author Contributions

MA drafted the manuscript, FU, AS, MOK and TA reviewed and analyzed the manuscript critically for technical aspects and mistakes.

## Conflict of Interest

The authors declare that the research was conducted in the absence of any commercial or financial relationships that could be construed as a potential conflict of interest.
